# Measuring Cognitive Load in Embodied Learning Settings

**DOI:** 10.3389/fpsyg.2017.01191

**Published:** 2017-08-02

**Authors:** Alexander Skulmowski, Günter Daniel Rey

**Affiliations:** Psychology of Learning with Digital Media, Chemnitz University of Technology Chemnitz, Germany

**Keywords:** embodied cognition, learning, cognitive load theory, cognitive load, measurement

## Abstract

In recent years, research on embodied cognition has inspired a number of studies on multimedia learning and instructional psychology. However, in contrast to traditional research on education and multimedia learning, studies on embodied learning (i.e., focusing on bodily action and perception in the context of education) in some cases pose new problems for the measurement of cognitive load. This review provides an overview over recent studies on embodied learning in which cognitive load was measured using surveys, behavioral data, or physiological measures. The different methods are assessed in terms of their success in finding differences of cognitive load in embodied learning scenarios. At the same time, we highlight the most important challenges for researchers aiming to include these measures into their study designs. The main issues we identified are: (1) Subjective measures must be appropriately phrased to be useful for embodied learning; (2) recent findings indicate potentials as well as problematic aspects of dual-task measures; (3) the use of physiological measures offers great potential, but may require mobile equipment in the context of embodied scenarios; (4) meta-cognitive measures can be useful extensions of cognitive load measurement for embodied learning.

## Embodied Learning Scenarios and Cognitive Load

In response to the findings concerning the influence of bodily perception, activity, and the physical environment on cognition in the fields of cognitive psychology and neuroscience (i.e., embodied cognition; Barsalou, 1999, 2003, 2008; Wilson, 2002; Glenberg, 2010; Shapiro, 2011), researchers investigating multimedia learning have begun to transfer insights gained from more basic embodiment research into applied settings (Paas and Sweller, 2012; Choi et al., 2014). Research on *embodied learning* usually focuses on the application of principles derived from embodiment research to the presentation of learning contents in educational settings (van Gog et al., 2009; Lindgren and Johnson-Glenberg, 2013).

A large part of educational research in the area of multimedia learning is concerned with learning settings in which, among others, the influence of visual and auditive design characteristics of learning materials is investigated (see Mayer, 2005, for an overview). However, formats of online education have been referred to as “disembodied” (Dall’Alba and Barnacle, 2005, p. 730). Still, even studies that include only very basic forms of bodily involvement and action beyond standard user interfaces are currently presented as being linked to embodiment research (e.g., Agostinho et al., 2015; Dubé and McEwen, 2015). Though, a large number of studies focusing on embodied learning deal with more sophisticated learning scenarios involving technology such as tangible user interfaces (e.g., Pouw et al., 2016a; Skulmowski et al., 2016) and mixed reality environments (e.g., Johnson-Glenberg et al., 2014, 2016). Therefore, the term *embodied learning scenarios* currently needs to be interpreted broadly (see Johnson-Glenberg et al., 2014, for a taxonomy).

Clark (1999, p. 348) introduced the theoretical distinction between “simple embodiment” and “radical embodiment”; Clark (1999) characterizes the former type of studies as embodied cognition research dealing with theoretical entities such as mental representation and computation, whereas the latter type of embodiment theory is described by Clark (1999) as rejecting these concepts (for further overviews, see Gallagher, 2005; Lindblom, 2015). However, educational researchers incorporating insights from embodied cognition usually do not exclusively align with the theoretical assumptions of either of these two views of embodiment. Thus, embodied learning currently needs to be considered to be a rather broad term. It includes studies based on the notion of multimodal mental representations related to Barsalou’s (1999) model (see Skulmowski et al., 2016, for an example), as well as theoretical assumptions derived from non-representational accounts focusing on the bodily enactment of learning contents (for overviews on enactivism in the context of learning, see Gallagher and Lindgren, 2015; Hutto et al., 2015; see Lindgren et al., 2016, for a study that exemplifies the enactive approach).

When applied to educational settings involving the measurement of cognitive load during learning, these theoretical models are operationalized in a multitude of ways. Interventions based on embodiment have been introduced to a large number of subjects and educational contexts, including physics (e.g., Johnson-Glenberg et al., 2014, 2016; Pouw et al., 2016a), language learning (Post et al., 2013), mathematics (e.g., Ruiter et al., 2015), and reading comprehension (e.g., Glenberg et al., 2004).

As a number of recent embodiment studies in the field of multimedia learning have revealed negative results concerning the effectiveness of body-based (and in some cases activity-based) forms of instruction (e.g., Post et al., 2013; Song et al., 2014; see Tran et al., 2017), a closer look at the cognitive mechanisms relevant to embodied learning seems warranted. The field of multimedia learning research employs a wide array of measures in order to assess the cognitive demands that learning materials impose on learners (see Brünken et al., 2003, for an overview). Cognitive load theory (CLT; Sweller, 1988; Sweller et al., 1998) is considered to have exhibited a major influence on the field of learning and instruction (Ozcinar, 2009); therefore, a large part of educational research concerned with embodied learning relates the findings of embodied cognition research to CLT (e.g., Paas and Sweller, 2012; Skulmowski et al., 2016). Conversely, progress in the field of cognitive load measurement is regarded to be important for the future of CLT (Paas et al., 2003). The objective of this review is to present subjective, behavioral, and physiological measurements of cognitive load in the context of embodied learning scenarios and to provide an assessment concerning the success of these instruments in recent studies.

## Cognitive Load Theory

The CLT model is built upon the premise that cognitive capacity is inherently limited by the availability of working memory resources (Sweller et al., 1998), based on the working memory model introduced by Baddeley (1992). CLT has been conceived as a computationalist framework right from the beginning (Sweller, 1988), implying a theoretical alignment with “simple embodiment” as defined by Clark (1999, p. 348).

In order to achieve an optimal exploitation of resources, CLT suggests interventions aimed at manipulating cognitive load, which is theoretically subdivided into three types of cognitive load (Sweller et al., 1998). These load types are described in the following section.

### Load Types

CLT researchers subscribe to a model dividing learners’ cognitive resources into three distinct kinds of cognitive load, namely the components *intrinsic load, extraneous load*, and *germane load* (Sweller et al., 1998). Intrinsic load is defined as measuring the inherent difficulty of learning contents (Sweller, 1994; Sweller and Chandler, 1994). Instructional factors concerning the design of learning materials are thought to influence the second component of CLT, the so-called extraneous load (Sweller, 1994; Sweller et al., 1998). CLT models usually include a third component, namely germane load, that is thought to be associated with the generation of knowledge structures in long-term memory (Sweller et al., 1998). Some researchers suggest to consider germane load as being linked to meta-cognitive processes (Valcke, 2002; Young et al., 2016). Moreover, there has been a debate around the issue whether it is actually necessary to distinguish between three types of cognitive load that called into doubt several assumptions made regarding germane load (de Jong, 2010; Kalyuga, 2011). Whelan (2007) reviewed several neuroscientific studies on learning and provided interpretations regarding the neural underpinnings of CLT.

### Cognitive Load Measurement for Embodied Learning

The measurement of cognitive load is generally regarded as a difficult task (e.g., de Jong, 2010; Martin, 2014), with some researchers even arguing against the use of distinct measures for the three load types (Kalyuga, 2011). In the following we will present and review methods that have been used to measure cognitive load in embodied learning scenarios, evaluate their success and highlight new developments.

#### Subjective Methods

One method of cognitive load measurement is the use of subjective scales (Paas et al., 2003). One commonly used question item developed by Paas (1992) asks participants for an indication of their *mental effort* during a learning task. In the following, we will provide some examples of recent embodiment studies that used this item:

Castro-Alonso et al. (2015a) found significant differences for the item developed by Paas (1992) when comparing animated learning materials with static depictions in the context of a brick construction task. Significant results of this item (Paas, 1992) indicating less cognitive load for static forms of instruction compared to animations were found in one trial of a related study (Experiment 1 in Wong et al., 2015). In contrast, other embodiment studies did not reveal significant effects using variants of this item (e.g., Ruiter et al., 2015; Pouw et al., 2016a). The mental effort item developed by Paas (1992) has been used in various studies to compute the *instructional efficiency* (Paas and van Merriënboer, 1993) of learning interventions (e.g., Castro-Alonso et al., 2014).

Another method of survey-based cognitive load measurement is the NASA Task Load Index (NASA-TLX; Hart and Staveland, 1988), which was recently used in studies concerning embodied learning (Skulmowski and Rey, 2017) and problem-solving (Kaspar and Vennekötter, 2015). The NASA-TLX survey contains of six question items: mental demand, physical demand, temporal demand, performance, effort, and frustration. Neither in the two studies reported by Skulmowski and Rey (2017) nor in the two experiments described by Kaspar and Vennekötter (2015) did the NASA-TLX result in significant differences concerning most of the cognitive variables of the NASA-TLX. The variable effort was significantly affected by the embodiment manipulation of sensing weight during a problem-solving task in Experiment 1 of Kaspar and Vennekötter (2015); the variable physical demands was significantly affected by the embodiment manipulation targeted at increasing physical exertion during a word learning task in both experiments of Skulmowski and Rey (2017). For a comparison between the NASA-TLX and the mental effort scale developed by Paas (1992), see Naismith et al. (2015).

Recently, cognitive load surveys measuring the three distinct load types were presented (e.g., Eysink et al., 2009; Leppink et al., 2013). The cognitive load survey instrument presented by Leppink et al. (2013) contains question items aimed at measuring intrinsic, extraneous, and germane load. However, the question items of this instrument (Leppink et al., 2013, p. 1070) refer to “instructions”, “explanations”, as well as “concepts” and “definitions”, suggesting that this survey may not be the optimal choice for instructional settings that rely less strongly on verbal instructions.

Another cognitive load survey was developed by Eysink et al. (2009) and contains one question item aimed at measuring intrinsic cognitive load, three items targeted at extraneous load, one item for germane load, and a last item for overall load. Eysink et al. (2009) used their cognitive load survey in order to measure cognitive load in the context of learning with (interactive) simulations (see Plass et al., 2009, for an overview of research on simulations). Hence, due to the links between research on interactive learning media and embodiment that have been suggested in the literature (see Lindgren and Johnson-Glenberg, 2013; Castro-Alonso et al., 2015b), one may assume that this survey could be appropriate for embodied learning research. Skulmowski et al. (2016) used this survey in a study investigating the effects of different interaction designs on learning. The study (Skulmowski et al., 2016) revealed a correspondence of the extraneous load ratings with retention scores (i.e., higher retention scores when extraneous load was lower and vice versa), providing evidence for the appropriateness of this questionnaire in embodied learning scenarios.

In summary, subjective measures may provide useful for cognitive load measurement in embodied scenarios if an appropriate survey is chosen. However, there are some general problems and theoretical issues related to the use of cognitive load questionnaires (see also de Jong, 2010). Van Gog and Paas (2008) highlight the problem that different phrasings in cognitive load question items might lead to results that may not be comparable. Furthermore, Leppink et al. (2013) suggest to conduct additional research specifically aimed at determining how participants understand cognitive load question items in different contexts. Lastly, Skulmowski et al. (2016) propose to subdivide extraneous load into more fine-grained components in the context of embodiment.

#### Behavioral Measures of Cognitive Load

Behavioral measures of cognitive load are an alternative to subjective measures (see Brünken et al., 2003, for an overview). In recent embodiment studies, a variety of behavioral measures have been utilized. For instance, Pouw et al. (2016a) included an analysis of reaction times alongside mental effort questions and performance measures. Dubé and McEwen (2015) used measures of response latency to investigate behavioral aspects of touchscreen interaction types in the context of embodied learning. Eye movements were used as an indicator of cognitive activity in a study presented in Pouw et al. (2016b). As the study (Pouw et al., 2016b) involved bodily activity in the form of gesturing, a mobile eye tracker was used.

Research on multimedia learning has made use of dual-task performance as a measure of cognitive load (e.g., Brünken et al., 2002). The procedure developed by Park and Brünken (2015) has been suggested for use in embodied learning research (Pouw et al., 2016c). However, recent findings suggest that specific types of dual-tasks may more strongly negatively affect performance in text-based forms of instruction compared to learning materials that additionally include pictures (van Genuchten et al., 2014). Kirschner et al. (2011) argue that the dual-task method may not be adequate for more elaborate settings. Therefore, we think that further research should be conducted to assess how dual-task measurements affect different cognitive processes involved in embodied learning scenarios.

#### Physiological Measures of Cognitive Load

Physiological measures of cognitive load (for overviews, see Brünken et al., 2003; Paas et al., 2003) include electroencephalography (EEG; e.g., Antonenko et al., 2010), heart rate (e.g., Paas et al., 1994), and pupil dilation (e.g., van Gerven et al., 2004). However, the application of brain imaging to embodied scenarios involving extensive movement may also introduce new difficulties (Gramann et al., 2014); in some cases requiring complex setups (e.g., Ehinger et al., 2014). On the other hand, EEG recordings can now be generated using low-budget EEG headsets (e.g., Debener et al., 2012; Palermo et al., 2017).

Pupil dilation was used in a variety of studies to measure cognitive load during learning and related cognitive tasks (e.g., van Gerven et al., 2004; Mitra et al., 2016). Recent research supports the idea that pupil dilation may be a valuable measure of mental demands in the context of movement-related studies (Jiang et al., 2015). Again, it should be noted that embodied learning scenarios in which participants perform movements may require the use of specialized hardware in the form of mobile eye tracking devices (e.g., Pouw et al., 2016b). As an increasing number of embodied learning scenarios are presented using immersive virtual reality equipment and related technologies (for a meta-analysis, see Merchant et al., 2014), pupillometric measurements may be obtained using eye trackers integrated into head-mounted displays (e.g., Skulmowski et al., 2014). In addition, other non-invasive physiological measures such as functional near-infrared spectroscopy were recently used in the context of embodied learning (Brucker et al., 2015).

## Outlook and Conclusion

In this review we have summarized the most widely used methods of cognitive load measurement as they pertain to embodied learning scenarios. Yet, several developments within the field of cognitive load measurement should be taken into greater account when investigating embodied learning. Repeated-measures study designs have been revealed to provide more appropriate measurements of cognitive load (e.g., van Gog et al., 2012; see Leppink and van Merriënboer, 2015), but only few studies on embodied learning have so far implemented repeated measurements of cognitive load (see Wong et al., 2015, for an example).

A number of recent studies on embodied learning employed meta-cognitive ratings in the form of judgments of learning, i.e., predictive self-assessments on how well one will be able to recall learning contents (e.g., Alban and Kelley, 2013; Skulmowski and Rey, 2017; for an overview on metacognition, see Dunlosky and Metcalfe, 2009). A study investigating the effects of a drawing activity on learning revealed that judgments of learning are even better predictors of learning results than cognitive load measurements (Schleinschok et al., 2017). Theoretical advances concerning embodied cognition have focused on the aspect of prediction (e.g., Clark, 2013, 2015) and there have been suggestions toward emphasizing meta-cognitive judgments within CLT (see Valcke, 2002; Skulmowski et al., 2016).

Judging from recent research on embodied learning, we can draw a number of conclusions for cognitive load measurement. Considering the reviewed studies utilizing subjective methods, cognitive load surveys appear to be a viable choice for measuring cognitive load in embodied learning. Yet, the different wordings found across different cognitive load surveys may pose a difficulty for choosing an appropriate survey for learning settings based on embodiment theory (see Subjective Methods).

Behavioral and physiological measures of cognitive load are objective alternatives to subjective cognitive load surveys (Brünken et al., 2003; Paas et al., 2003). In Section “Behavioral Measures of Cognitive Load” we have presented arguments in favor and against the use of dual-task performance as cognitive load measurement. From the reviewed research on physiological measures, we can see an enormous potential for these types of measures for educational and applied research based on embodied cognition. However, embodied learning may require specialized equipment allowing to perform mobile recordings (see Physiological Measures of Cognitive Load).

To conclude, researchers interested in embodied learning have a wide variety of tools for cognitive load measurement at their disposal. Yet, as we have seen, some methods are more appropriate than others for specific situations. Therefore, further research is necessary to determine more detailed guidelines regarding the use of cognitive load measurement methods in embodied scenarios.

## Author Contributions

AS created the initial draft of the manuscript. GR provided critical revisions to the draft. Both authors read and approved the final manuscript.

## Conflict of Interest Statement

The authors declare that the research was conducted in the absence of any commercial or financial relationships that could be construed as a potential conflict of interest.
